# *Drosophila* TRF2 and TAF9 regulate lipid droplet size and phospholipid fatty acid composition

**DOI:** 10.1371/journal.pgen.1006664

**Published:** 2017-03-08

**Authors:** Wei Fan, Sin Man Lam, Jingxue Xin, Xiao Yang, Zhonghua Liu, Yuan Liu, Yong Wang, Guanghou Shui, Xun Huang

**Affiliations:** 1 State Key Laboratory of Molecular Developmental Biology, Institute of Genetics and Developmental Biology, Chinese Academy of Sciences, Beijing, China; 2 University of Chinese Academy of Sciences, Beijing, China; 3 Academy of Mathematics and Systems Science, National Center for Mathematics and Interdisciplinary Sciences, Chinese Academy of Sciences, Beijing, China; Stanford University School of Medicine, UNITED STATES

## Abstract

The general transcription factor TBP (TATA-box binding protein) and its associated factors (TAFs) together form the TFIID complex, which directs transcription initiation. Through RNAi and mutant analysis, we identified a specific TBP family protein, TRF2, and a set of TAFs that regulate lipid droplet (LD) size in the *Drosophila* larval fat body. Among the three *Drosophila* TBP genes, *trf2*, *tbp* and *trf1*, only loss of function of *trf2* results in increased LD size. Moreover, TRF2 and TAF9 regulate fatty acid composition of several classes of phospholipids. Through RNA profiling, we found that TRF2 and TAF9 affects the transcription of a common set of genes, including peroxisomal fatty acid β-oxidation-related genes that affect phospholipid fatty acid composition. We also found that knockdown of several TRF2 and TAF9 target genes results in large LDs, a phenotype which is similar to that of *trf2* mutants. Together, these findings provide new insights into the specific role of the general transcription machinery in lipid homeostasis.

## Author summary

Lipid droplets (LD) are main lipid storage structures in most cells. The size of LDs varies greatly in different cell types or different metabolic states to accommodate cellular functions and metabolism demands. How cells regulate the lipid storage and LD dynamics is not fully understood. Here, we identified that general transcription factors, including a specific TBP (TATA-box binding protein) family protein TRF2 (TBP-related factor 2) and several TAFs (TBP-associated factors), regulate LD size in the fruitfly larval fat body. Moreover, quantitated lipid analysis reveals that TRF2 and TAF9 affect the fatty acid composition of several classes of phospholipids. We showed that TRF2 and TAF9 regulate transcription of several target genes, including peroxisomal fatty acid β-oxidation-related genes which likely mediate the effect of TRF2 and TAF9 on phospholipid fatty acid composition. We also found that overexpression of some target genes restores the LD phenotype in *trf2* mutants. Our findings therefore reveal specific roles of general transcription factors in lipid homeostasis.

## Introduction

The Pol II (RNA polymerase II)-GTF (general transcription factor)-Mediator-TF (transcription factor)-Effector (target genes) axis of eukaryotic transcriptional regulation has been well established in many biological processes. Biochemical, cellular and physiological studies have discovered that adipocyte differentiation and lipid homeostasis in adipose tissue are regulated by an adipogenic transcription cascade including C/EBPs and PPARs, a lipogenic enzymatic cascade and an increasing list of specific transcription factors such as SREBP, LXR, FXR, NHR-49 and HNF4α [1–3]. Previous studies also revealed that by interacting with specific transcription factors, general transcription machineries, including the Mediator subunits MED1, MED13, MED14, MED15, MED23 and MED25, play important roles in lipid metabolism [4–10]. The Mediator complex bridges general transcription factors and Pol II to specific transcription factors to regulate transcription. However, it is not fully understood whether general transcription factors exhibit specificities in regulating lipid metabolism.

Adipocytes store neutral lipids in lipid droplets (LDs), which are intracellular organelles consisted of a monolayer of phospholipids, a neutral lipid core, and associated proteins [11]. The size of LDs varies greatly in different cell types to accommodate distinct cellular functions. White adipocyte usually contains a large unilocular LD for lipid storage, while brown adipocyte has many small LDs for rapid lipolysis. It is well known that the content of the lipid core, the composition of monolayer phospholipids and the protein machinery for LD fusion affect the size of LDs [12–16]. In addition, taking advantage of genetic and cell based-RNAi screens, systematic studies in *C*. *elegans* and *Drosophila* cultured cells by lipid staining and/or imaging, and in *Drosophila* adults by measuring total levels of triacylglycerol (TAG) have identified numerous genes and cellular pathways involved in the regulation of lipid storage and LD dynamics [17–21]. Along with other functional studies [22–26], these findings provide valuable clues as to the complicated regulation of lipid storage and LD dynamics. However, our understanding of the lipid storage network and regulation of LD dynamics is far from clear.

In this study, we identified that several components of the general transcription factor TFIID complex, including a specific TBP (TATA-box binding protein) family protein TRF2 (TBP-related factor 2) and several TAFs (TBP-associated factors), regulate LD size in the *Drosophila* larval fat body, which is the adipose tissue in flies. Unlike TBP, which binds to TATA-containing promoters and initiates Pol II-dependent gene transcription, TRF2 acts as a core promoter-selective factor and regulates transcription from TATA-less promoters [27–30]. In *Drosophila*, *trf2* is required for several specific biological processes such as embryonic development, germ cell differentiation and metamorphosis [31, 32].

*Drosophila* has three TBP genes and only loss of function of *trf2* results in large LD phenotype. Moreover, lipidomic analysis reveals that TRF2 and TAF9 also affect the fatty acid composition of several classes of phospholipids. We showed that TRF2 and several core TAFs affect transcription of several target genes related to lipid metabolism. The regulatory effect of TRF2 and TAF9 on phospholipid fatty acid composition is most likely mediated by genes involved in the peroxisomal fatty acid β-oxidation. We also found that overexpression of some target genes restores the LD phenotype in *trf2* mutants. Therefore, our study reveals specific roles of general transcription factors, namely TRF2 and TAF9, in lipid homeostasis.

## Results

### TAF9 affects *Drosophila* fat body LD size

**Fig 1:**
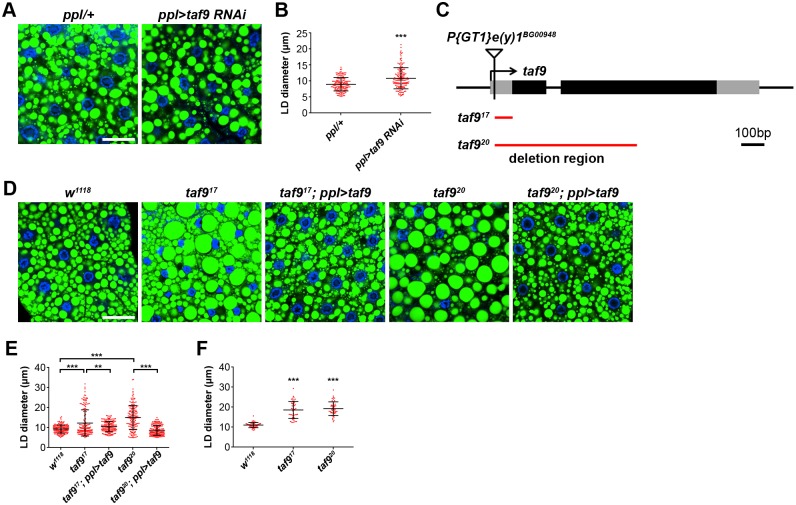
TAF9 is required for LD size regulation in *Drosophila* fat body. (A) Knockdown of *taf9* causes enlarged LDs in larval fat body. LDs were stained by BODIPY (green) and nuclei were stained by DAPI (blue). Scale bar represents 50μm. (B) Quantification of LD diameter in (A). Each point represents a single LD. Error bars represent ±SD. ***: p < 0.001. (C) The genomic structure of *taf9*. Black boxes represent coding regions and grey boxes represent un-translated regions. The positions of the transposon insertion *e(y)1*^*BG00948*^, the 79bp deletion of *taf9*^*17*^ and the 627bp deletion of *taf9*^*20*^ are indicated. (D) BODIPY staining of LDs in 3^rd^ instar larval fat bodies of different genetic backgrounds. *taf9*^*17*^ and *taf9*^*20*^ mutants have large LDs. Expression of *taf9* using the fat body-specific driver *ppl-Gal4* fully rescues the mutant phenotype. Nuclei were stained by DAPI (blue). Scale bar represents 50 μm. (E) Quantification of LD diameter in (D). Data were analyzed by one-way ANOVA with Tukey’s multiple comparisons test. Error bars represent ±SD. ***: p < 0.001; **: p < 0.01. (F) Quantification of the diameter of the three largest LDs per cell in 16 cells from *w*^*1118*^, *taf9*^*17*^ and *taf9*^*20*^ mutants. Data were analyzed by one-way ANOVA with Dunnett’s multiple comparisons test. Error bars represent ±SD. ***: p < 0.001.

To systematically identify genes that regulate lipid storage in adipose and non-adipose tissues, we used the tissue-specific *Gal4-UAS* system to perform an RNAi screen in *Drosophila* larval fat body and salivary gland with *ppl-Gal4* [33]. We found that knockdown of the general transcription factor TFIID complex component TAF9 results in enlarged LDs in the fat body (Fig 1A). The diameter of LDs stained with BODIPY dye was measured and quantified. In *ppl-Gal4* controls, the average size of LDs is around 9 μm and the largest is around 14 μm. In *taf9* RNAi fat body, the average size of LDs increases to around 11 μm and the largest is over 20 μm (Fig 1B).

To confirm the results from RNAi, we examined the *taf9* mutant phenotype. *taf9* is also known as *enhancer of yellow 1*, *e(y)1*, and was originally reported to affect the Yellow phenotype [34]. In another study, *taf9* partial loss-of-function mutants were found to impair female fertility and oogenesis [35]. Furthermore, *taf9* was reported to be involved in the transcriptional regulation of Notch signaling [36]. We generated two N-terminal deletion mutants of *taf9* through P-element imprecise excision (Fig 1C). Both mutants are homozygous lethal after the wandering 3^rd^ instar larval stage. A few of mutant larvae can form white prepupa and then die. We therefore examined the LD phenotype in the fat body of active wandering 3^rd^ instar mutant larvae. In *taf9*^*17*^ fat bodies, the average size of LDs increases to around 12 μm and the largest one is over 30 μm. In *taf9*^*20*^ fat bodies, the average size of LDs is around 15 μm and the largest is over 34 μm (Fig 1D and 1E). To more specifically describe the large LD phenotype, we quantified the size of three largest LDs in individual fat cells. In wild type, the average size of the largest LDs is around 11 μm, while in *taf9*^*17*^ and *taf9*^*20*^ mutants, it increases to around 19 μm (Fig 1F). These results are consistent with the *taf9* RNAi results. We also examined the phenotype of *taf9*^*17*^ mutants at both 2^nd^ and early 3^rd^ instar larval stages. There was no significant difference in LD size between *taf9*^*17*^ mutants and controls (S1 Fig).

To further validate the mutant phenotype, we next performed rescue experiments. The large LD phenotype of *taf9*^*17*^ and *taf9*^*20*^ mutants was rescued by overexpressing *taf9* in the fat body using *ppl-Gal4*, indicating that *taf9* functions autonomously in fat body to regulate LD size (Fig 1D and 1E). Together, the phenotypic analysis of RNAi knockdown animals and P-element-derived knockout mutants indicate that *taf9* plays an important role in LD size regulation in the fat body.

### TFIID core complex genes are required for LD size regulation

**Fig 2:**
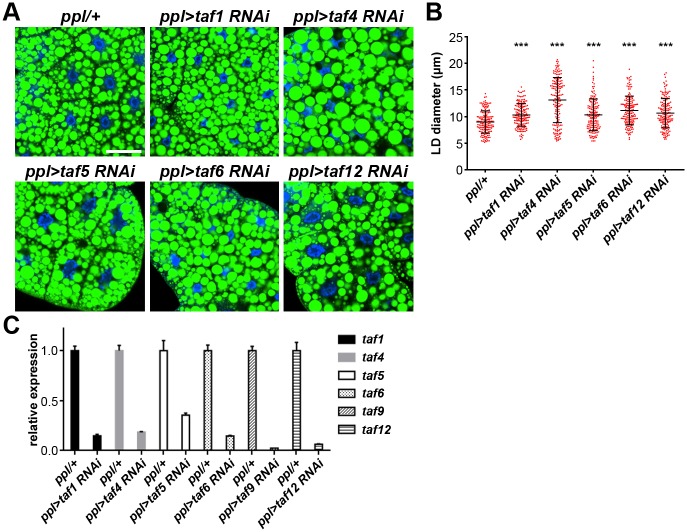
TFIID core complex components are required for LD size regulation. (A) Knockdown of *taf1*, *taf4*, *taf5*, *taf6* and *taf12* results in enlarged LDs in larval fat bodies. The RNAi strains of *taf1* and *taf6* used here are KK100418 and KK101106, respectively. Scale bar represents 50 μm. (B) Quantification of LD diameter in (A). Data were analyzed by one-way ANOVA with Dunnett’s multiple comparisons test. Error bars represent ±SD. ***: p < 0.001. (C) Relative mRNA levels of *taf* genes in fat bodies from different backgrounds. Error bars represent ±SD.

TAF9 is one subunit of TFIID, a multi-subunit complex composed of TBP and TAFs. TAF4, TAF5, TAF6, TAF9 and TAF12 form a functional core subcomplex within TFIID [37]. To investigate the involvement of the other TFIID core TAFs in LD size regulation, we knocked them down by crossing available RNAi lines to *ppl-Gal4*. The fat body expression of these *taf* genes and knockdown efficiency were examined by qRT-PCR. As expected, the expression levels of these genes decreased significantly in the fat body with RNAi (Fig 2C). Phenotypically, RNAi of *taf4*, *taf5*, *taf6* and *taf12* all result in enlarged LDs in the fat body (Fig 2A and 2B and S1 Table). For example, in *taf4* RNAi fat body, the average size of LDs increases to around 13 μm and the largest is over 20 μm. These results suggest a general requirement for the TFIID core complex in LD size regulation. Moreover, knockdown of *taf1*, which is a non-core complex component, also leads to a large LD phenotype (Fig 2A and 2B). Knockdown of other non-core complex components did not affect LD size (S1 Table). It is possible that either they are not required in this context or the RNAi efficiencies were not high enough to cause a measurable LD phenotype.

### Among the three TBP family proteins, TRF2 is specifically involved in LD size regulation

**Fig 3:**
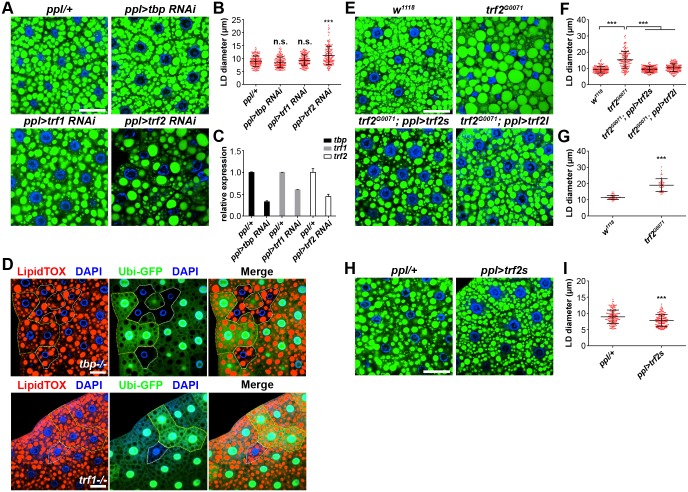
Among three TBP family members in *Drosophila*, only TRF2 specifically regulates LD size. (A) Knockdown of *trf2* in larval fat body leads to increased LD size whereas knockdown of *tbp* or *trf1* has no significant phenotype. Scale bar represents 50 μm. (B) Quantification of LD diameter in (A). Data were analyzed by one-way ANOVA with Dunnett’s multiple comparisons test. Error bars represent ±SD. ***: p < 0.001; n.s.: nonsignificant. (C) Relative mRNA levels of *tbp*, *trf1* and *trf2* in fat bodies from different backgrounds. Error bars represent ±SD. (D) Analysis of *tbp* and *trf1* mutant fat cell clones. Mutant fat cells (GFP-negative) are marked by white dashed lines and twin-spot control cells (GFP-positive) are outlined by yellow dashed lines. LDs were stained by LipidTOX (red) and nuclei were stained by DAPI (blue). There is no difference between *tbp* or *trf1* mutant fat cells and control fat cells. Scale bar represents 50 μm. (E) BODIPY staining of LDs in 3^rd^ instar larval fat bodies of different genetic backgrounds. *trf2*^*G0071*^ mutants have an enlarged LD phenotype, which is fully rescued by expression of either *trf2l* or *trf2s* in the fat body. Scale bar represents 50 μm. (F) Quantification of LD diameter in (E). Data were analyzed by one-way ANOVA with Tukey’s multiple comparisons test. Error bars represent ±SD. ***: p < 0.001. (G) Quantification of the diameter of the three largest LDs per cell of *w*^*1118*^ and *trf2*^*G0071*^ mutants. Error bars represent ±SD. ***: p < 0.001. (H) Overexpression of *trf2s* results in small LDs in larval fat body. Scale bar represents 50 μm. (I) Quantification of LD diameter in (H). Error bars represent ±SD. ***: p < 0.001.

How do the aforementioned TAF proteins affect LD size? TAF proteins associate with TBP to form the TFIID complex, which binds to the core promoter and initiates assembly of the PIC (preinitiation complex) to facilitate Pol II-dependent transcription initiation. There are three TBP homologs in *Drosophila*: the founding member TBP, a closely related member TRF1, and a distantly related member TRF2 [38, 39]. TBP and TRF2 exist in all metazoans; TRF1 is specific to insects, while vertebrates have TRF3. TBP binds to the TATA box, while TRF2 was reported to recognize TATA-less promoters. We firstly knocked down all three TBP family members (*tbp*, *trf1* and *trf2*) in the fat body by RNAi. Only knockdown of *trf2* results in increased LD size: the average size of LDs increases to around 11 μm and the largest is over 22 μm (Fig 3A and 3B). This phenotype is reminiscent of that generated by knockdown of *taf* genes. Since the expressions of all three genes in the fat body are reduced by RNAi (Fig 3C), these data suggest that the TFIID core complex mediates TRF2-dependent transcription to regulate LD size. To further investigate whether *trf2* also regulates LD size in adult stage, we knocked down *trf2* in adult fat body with *cg-Gal4*. Knockdown of *trf2* leads to more small LDs compared to controls (S2 Fig), while knockdown of *taf9* causes lethality at pupal stage. These results indicate that TRF2 plays an important role to regulate LD size in both larval and adult stages.

To further verify our findings with *trf2* RNAi, we next investigated the fat body phenotype of *tbp*, *trf1* and *trf2* mutants. *tbp*^*f00190*^ is a piggyBac insertion mutation while *trf1*^*GS16912*^ is a P-element insertion mutation in the first exon. Since both *tbp* and *trf1* mutants are lethal at early developmental stages [40], we generated mutant fat cell clones to examine their LD phenotype. Both *tbp* and *trf1* mutant fat cells display a wild-type LD phenotype compared with the surrounding control cells, demonstrating that *tbp* and *trf1* are likely not involved in LD size regulation in the fat body (Fig 3D). The *trf2* mutant allele *trf2*^*G0071*^ has a P-element insertion in the intron and displays lethality during metamorphosis, allowing us to investigate the fat body phenotype in wandering 3^rd^ instar larvae. Similar to the RNAi results, the fat cells in *trf2*^*G0071*^ mutants also have large LDs: the average size is around 15 μm and the largest is over 30 μm (Fig 3E and 3F). When the three largest LDs in each fat cell are quantified, the average size of LDs in *trf2*^*G0071*^ mutants is around 19 μm (Fig 3G). These results indicate that TRF2 is specifically involved in LD size regulation. Moreover, *trf2*^*G0071*^ mutants have enlarged LDs in the fat body at both 2^nd^ and early 3^rd^ instar larval stages, indicating that TRF2 is also required for LD size regulation at early developmental stages (S1 Fig).

*Drosophila trf2* encodes two protein isoforms: a 632-amino-acid short isoform (TRF2s) containing the DNA binding domain, which shows similarity to the core domain of TBP; and a 1715-amino-acid long isoform (TRF2l) in which the identical short TRF2 sequence is preceded by a long N-terminal region [31]. To examine the contribution of different TRF2 isoforms to the LD phenotype observed in *trf2*^*G0071*^ mutants, we performed rescue experiments by expressing the individual isoforms. We found that the large LD phenotype of *trf2*^*G0071*^ mutants was rescued by expressing either TRF2 isoform in the fat body, indicating that the large LD phenotype is due to *trf2* deficiency and *trf2* plays an autonomous role in regulating LD size (Fig 3E and 3F). Furthermore, the rescue data reveal that the long N-terminal region of TRF2l is not required for the function of TRF2 in LD size regulation. In addition, overexpression of *trf2s* with *ppl-Gal4* at 29°C leads to small LDs. The average size of LDs is around 8 μm and the largest is around 13 μm (Fig 3H and 3I). Overexpression of *trf2l* with *ppl-Gal4* at 29°C leads to developmental arrest at early 3^rd^ instar larval stage. Together, our findings derived from phenotypic analysis of RNAi knockdown and mutant animals reveal a specific role of TRF2, but not TBP or TRF1, in LD size control.

### TRF2 and TAF9 regulate the fatty acid composition of phospholipids

**Fig 4:**
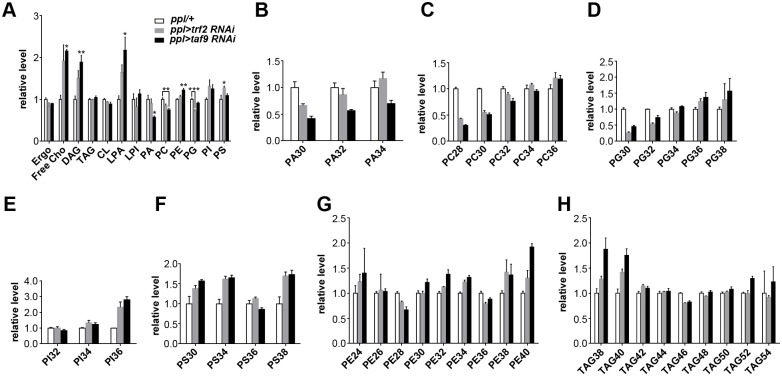
TRF2 and TAF9 regulate the phospholipid fatty acid composition. (A) The relative level (normalized to total phospholipids) of phospholipids and neutral lipids in the larval fat bodies of different backgrounds. Assays were done in triplicate. Data were analyzed by one-way ANOVA with Dunnett’s multiple comparisons test. Error bars represent ±SEM. ***: p < 0.001; **: p < 0.01; *: p < 0.05. Ergo, ergosterol; Free Cho, free cholesterol; DAG, diacylglycerol; TAG, triacylglycerol; CL, cardiolipin; LPA, lysophosphatidic acid; PA, phosphatidic acid; PC, phosphatidylcholine; PE, phosphatidylethanolamine; PG, phosphatidylglycerol; PI, phosphatidylinositol; PS, phosphatidylserine. (B-H) The relative level (normalized to total phospholipids) of species with different fatty acid chain lengths in PA (B), PC (C), PG (D), PI (E), PS (F), PE (G), and TAG (H) in the larval fat bodies of different backgrounds. Error bars represent ±SEM.

Previous studies have revealed that the content of neutral lipid core and/or the levels of monolayer phospholipids contribute to LD size. To reveal the effects of TRF2 and TAF9 on lipid composition, we profiled the level of phospholipids and neutral lipids in the fat body of *trf2* RNAi and *taf9* RNAi (Fig 4A). In *trf2* RNAi and *taf9* RNAi, the level of most phospholipids, when normalized to total phospholipids, does not change dramatically compared to the *ppl-Gal4* control. Phosphatidylinositol (PI) and phosphatidylserine (PS) are slightly increased while phosphatidylcholine (PC) and phosphatidylglycerol (PG) are slightly decreased in both *trf2* and *taf9* RNAi. Moreover, the TAG level is not changed in *trf2* and *taf9* RNAi when normalized to total phospholipids. In contrast, the levels of free cholesterol, diacylglycerol (DAG) and lysophosphatidic acid (LPA) are significantly increased in both *trf2* and *taf9* RNAi (Fig 4A).

We also compared the fatty acid composition of major phospholipids and TAG (Fig 4B–4H). We noticed changes in the phospholipid fatty acid chain length in *trf2* and *taf9* RNAi. Several classes of phospholipids in *trf2* and *taf9* RNAi tend to be composed of fatty acids with long chain lengths. When we compared the relative amounts of major phospholipids based on the total fatty acid chain length, there are more phospholipid species with longer fatty acid chains in *trf2* and *taf9* RNAi fat bodies compared with *ppl-Gal4* controls. This trend was obvious in phosphatidic acid (PA), PC, PG and PI but less significant in phosphatidylethanolamine (PE), PS and TAG (Fig 4B–4H). These results suggest that TRF2 and TAFs may regulate the expression of a specific set of genes which are important for controlling LD size and phospholipid fatty acid composition.

### Identification of *trf2* and *taf9* target genes

**Fig 5:**
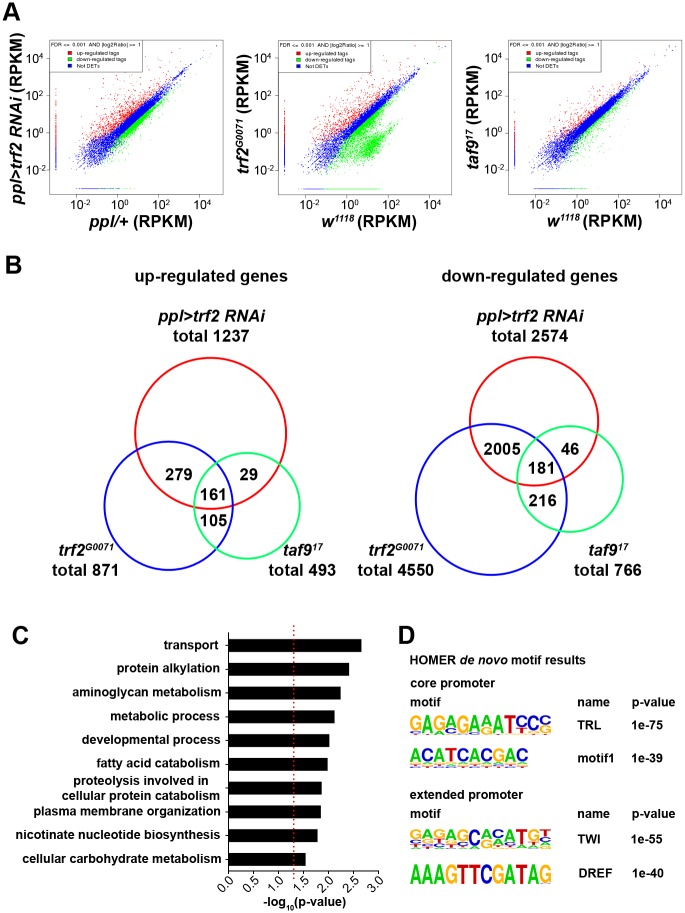
TRF2 and TAF9 are required for the expression of a set of target genes. (A) RNA-seq analysis of fat bodies from controls, *trf2* RNAi, *trf2*^*G0071*^ mutants and *taf9*^*17*^ mutants. Scatter plots show gene expression profiles of each group. Red dots represent up-regulated genes and green dots represent down-regulated genes. Blue dots represent genes below the cutoff. (B) Overview of the overlap of differentially expressed genes in *trf2* RNAi, *trf2*^*G0071*^ mutants and *taf9*^*17*^ mutants. Venn diagrams show up- and down-regulated genes in each group. (C) GO term analysis of 181 genes that are down-regulated in all three groups. The list shows the top ten GO term biological process categories ranked by p-value. The red dashed line represents p-value = 0.05. (D) HOMER motif analysis of promoters of the 181 genes. The list shows several top-scoring motifs in core promoters or extended promoters. The p-value represents the significance of the enrichment of the motifs.

To identify the potential target genes regulated by TRF2 and TAF9 in controlling LD size and phospholipid fatty acid composition, we performed RNA-seq to profile global gene expression patterns of the fat body in controls, *trf2* RNAi, *trf2*^*G0071*^ mutants and *taf9*^*17*^ mutants. We defined differentially expressed genes as showing more than twofold difference in gene expression and having a FDR (False Discovery Rate) cutoff of ≤ 0.001 (Fig 5A). Compared to the *ppl-Gal4* control, there are 2574 down-regulated genes and 1237 up-regulated genes in *trf2* RNAi (Fig 5B). In *trf2*^*G0071*^ mutants, 4550 genes are down-regulated and 871 genes are up-regulated compared to *w*^*1118*^ controls (Fig 5B). In both *trf2* RNAi and *trf2*^*G0071*^ mutant fat bodies, there are many more down-regulated genes than up-regulated genes, consistent with the positive role of *trf2* in gene transcription. The up-regulated gene expression in *trf2* RNAi or *trf2*^*G0071*^ mutants may be due to secondary effects. The fact that there are more down-regulated genes in *trf2*^*G0071*^ mutants than *trf2* RNAi may be caused by the background difference between *trf2*^*G0071*^ mutants and *w*^*1118*^ controls or differences in the strength of the loss of function between RNAi and mutants. Importantly, there is a significant overlap (2186/2574, 85%) of down-regulated genes between *trf2*^*G0071*^ mutants and *trf2* RNAi, indicating that the results are valid. For *taf9*^*17*^ mutants, we found 766 down-regulated genes and 493 up-regulated genes compared to *w*^*1118*^ controls (Fig 5B). Compared to gene expression changes in *trf2*^*G0071*^ mutants or *trf2* RNAi, we found fewer differentially expressed genes in *taf9*^*17*^ mutants, suggesting that *taf9* may only participate in some *trf2*-regulated transcription events. In addition, partial overlap (397/766, 52%) of down-regulated genes between *taf9*^*17*^ mutants and *trf2*^*G0071*^ mutants suggests that TAF9 also participates in transcription regulation mediated by other TBP proteins (Fig 5B).

Inactivation of either *trf2* or *taf9* causes similar LD phenotype, suggesting that they may have common target genes. Therefore, we next focused on the down-regulated genes that overlap between *trf2* RNAi, *trf2*^*G0071*^ and *taf9*^*17*^ mutants. 181 genes are down-regulated in all three groups, while 46 genes are only down-regulated in *taf9*^*17*^ mutants and *trf2* RNAi, and 216 genes are only down-regulated in *trf2*^*G0071*^ mutants and *taf9*^*17*^ mutants (Fig 5B). Gene ontology (GO) term analysis of the 181 genes shared by all groups showed enrichment of several categories of biological process, including metabolic processes involving various metabolites such as fatty acids, carbohydrates and aminoglycans. This suggests that *trf2* and *taf9* may play important roles in metabolic regulation in the fat body. Furthermore, there are also other significant GO term categories such as transport, protein alkylation and developmental processes (Fig 5C). We did not find obvious candidate genes that have been reported to cause large LD phenotypes, such as CCT or Plin1, suggesting that *trf2* and *taf9* may not directly regulate these genes.

We next investigated the regulatory sequence elements in the promoters of the genes that are regulated by TRF2 and TAF9. We performed an unbiased analysis of the promoters of the 181 genes to identify motifs associated with known TFs or *de novo* motifs. Core promoters (-100 to +50 relative to +1 of the transcription start site) and extended promoters (-500 to +50) were analyzed by HOMER program. A Trithorax-like (TRL)-binding motif and a new *de novo* motif1 are highly enriched in core promoters, while Twist (TWI)-binding motif and DREF (DRE-binding factor) motif are highly enriched in extended promoters (Fig 5D). These results suggest that TRF2 and TAF9 may cooperate with these transcription factors to regulate transcription of target genes. The significant enrichment of DREF binding motif in the extended promoters is consistent with previous studies showing that DREF exists in a multisubunit TRF2-containing complex [27] and that DRE (DNA replication-related element) is strongly associated with TRF2-bound promoters [28].

### TRF2 and TAF9 acts on genes related to peroxisomal fatty acid β-oxidation to regulate the fatty acid composition of phospholipids

**Fig 6:**
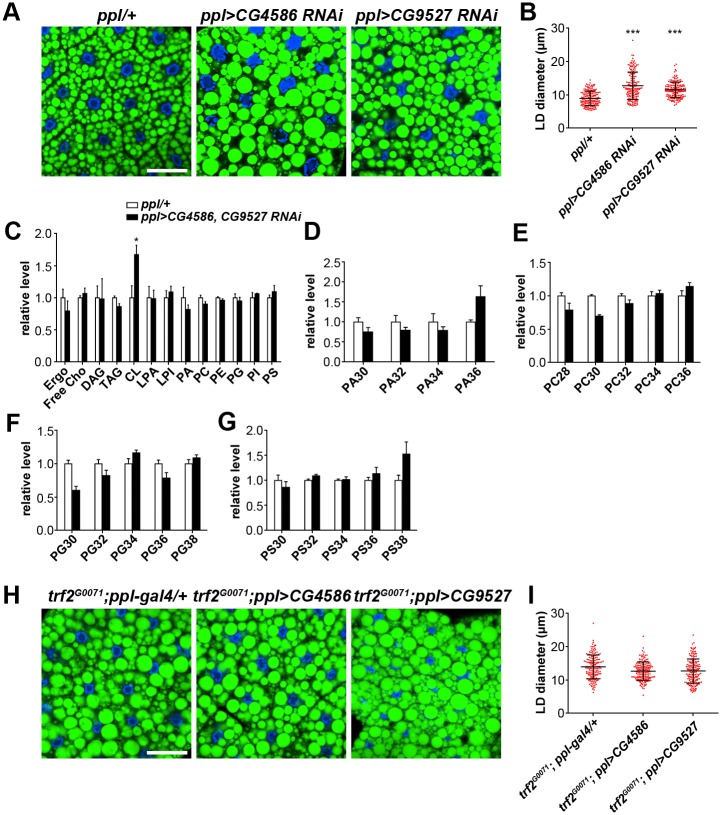
TRF2 and TAF9 regulate expression of peroxisomal fatty acid β-oxidation pathway genes. (A) Knockdown of *CG4586* and *CG9527* causes increased LD size in larval fat bodies. The RNAi strains of *CG4586* and *CG9527* used here are 4586R-2 and KK105295, respectively. Scale bar represents 50 μm. (B) Quantification of LD diameter in (A). Data were analyzed by one-way ANOVA with Dunnett’s multiple comparisons test. Error bars represent ±SD. ***: p < 0.001. (C) The relative level (normalized to total phospholipids) of phospholipids and neutral lipids in the larval fat bodies of *ppl-Gal4* and *CG4586*/*CG9527* double RNAi. Assays were done in triplicate. Error bars represent ±SEM. *: p < 0.05. (D-G) The relative level (normalized to total phospholipids) of species with different fatty acid chain lengths in PA (D), PC (E), PG (F), PS (G) in the larval fat bodies of different backgrounds. Error bars represent ±SEM. (H) BODIPY staining of LDs in larval fat bodies of different genetic backgrounds. Expression of *CG4586* and *CG9527* in the fat body only marginally rescues the *trf2*^*G0071*^ mutant phenotype. Scale bar represents 50 μm. (I) Quantification of LD diameter in (H). Error bars represent ±SD.

The GO term analysis of 181 potential TRF2 and TAF9 target genes identified two genes, *CG4586* and *CG9527*, belong to the GO term category of fatty acid catabolism. *CG4586* and *CG9527* encode ACOX (acyl-CoA oxidase) which is involved in peroxisomal fatty acid β-oxidation by converting acyl-CoA to *trans*-Δ^2^-enoyl-CoA [41]. In addition, two genes that are down-regulated in *trf2* RNAi and *taf9*^*17*^ mutants, *CG9149* and *CG9577*, encode β-ketoacyl-CoA thiolase and ECH (enoyl-CoA hydratase) respectively. Both of these enzymes are involved in peroxisomal fatty acid β-oxidation. Peroxisome β-oxidation generates medium-chain (C≤14) fatty acid-CoA by the catabolism of very long-chain (C≥22) fatty acids (VLCFAs) and some long-chain (C16-C20) fatty acids (LCFAs) in mammals [42]. In *C*. *elegans*, defects in the peroxisomal fatty acid β-oxidation pathway cause LD expansion associated with altered fatty acid composition in both total lipids and TAG and the supersized LD phenotype, reminiscent of the phenotype of *trf2*^*G0071*^ and *taf9*^*17*^ mutants [43]. We found that knockdown of either *CG4586* or *CG9527* leads to significantly increased LD size, although it is not as strong as in *trf2*^*G0071*^ or *taf9*^*17*^ mutants (Fig 6A and 6B). The LD phenotype was also confirmed with independent RNAi lines (S2 Table). Therefore, we further explored the function of *CG4586* and *CG9527*, and the connection between *trf2*/*taf9* and *CG4586*/*CG9527*.

There are six ACOX genes (*CG4586*, *CG5009*, *CG9527*, *CG9707*, *CG9709* and *CG17544*) in the *Drosophila* genome, among which only *CG4586* and *CG9527* have significantly enriched gene expression (4.9 fold and 3.2 fold respectively) in the larval fat body (FlyAtlas). Transcription of both *CG4586* and *CG9527* was dramatically reduced in the fat body of *trf2*^*G0071*^ and *taf9*^*17*^ mutants. In contrast, transcription of the other four ACOX genes was largely unaffected by *trf2* and *taf9* mutations based on our RNA-seq data.

Since ACOX functions in the homeostasis of VLCFAs, LCFAs and medium chain fatty acids, we further investigated the effects of *CG4586* and *CG9527* on lipid composition. We profiled the levels of phospholipids and neutral lipids in the fat body of *CG4586*/*CG9527* double RNAi through lipidomic analysis (Fig 6C). The levels of most phospholipids and neutral lipids, when normalized to total phospholipids, do not change remarkably compared to the *ppl-Gal4* control. Only cardiolipin (CL) is significantly increased in *CG4586*/*CG9527* double RNAi. Similar to the results in *trf2* and *taf9* RNAi, there are also changes in the phospholipid fatty acid composition in *CG4586*/*CG9527* double RNAi. In PA, PC, PG and PS, the relative amounts of phospholipid species with longer fatty acid chains are increased in *CG4586*/*CG9527* double RNAi fat bodies compared with *ppl-Gal4* controls (Fig 6D–6G). Together with the phospholipid profiling of *trf2* RNAi and *taf9* RNAi (Fig 4B–4G), these results suggest that TRF2 and TAF9 regulate the fatty acid composition of phospholipids likely by modulating the expression of peroxisomal fatty acid β-oxidation genes, such as *CG4586* and *CG9527*.

Next we explored the contribution of these two ACOX genes to the large LD phenotype in *trf2*^*G0071*^ mutant fat body. We performed the rescue experiments but found that overexpression of either these two genes in *trf2*^*G0071*^ mutant fat body only marginally rescued the large LD phenotype (Fig 6H and 6I). It is possible that the large LD phenotype in *trf2*^*G0071*^ mutants is not due to downregulated expression of these two ACOX genes. Alternatively, it is possible that the large LD phenotype is due to impairment of the whole peroxisomal fatty acid β-oxidation pathway, which cannot be restored by simply overexpressing one ACOX gene.

### *trf2* and *taf9* target genes are important for LD size regulation

**Fig 7:**
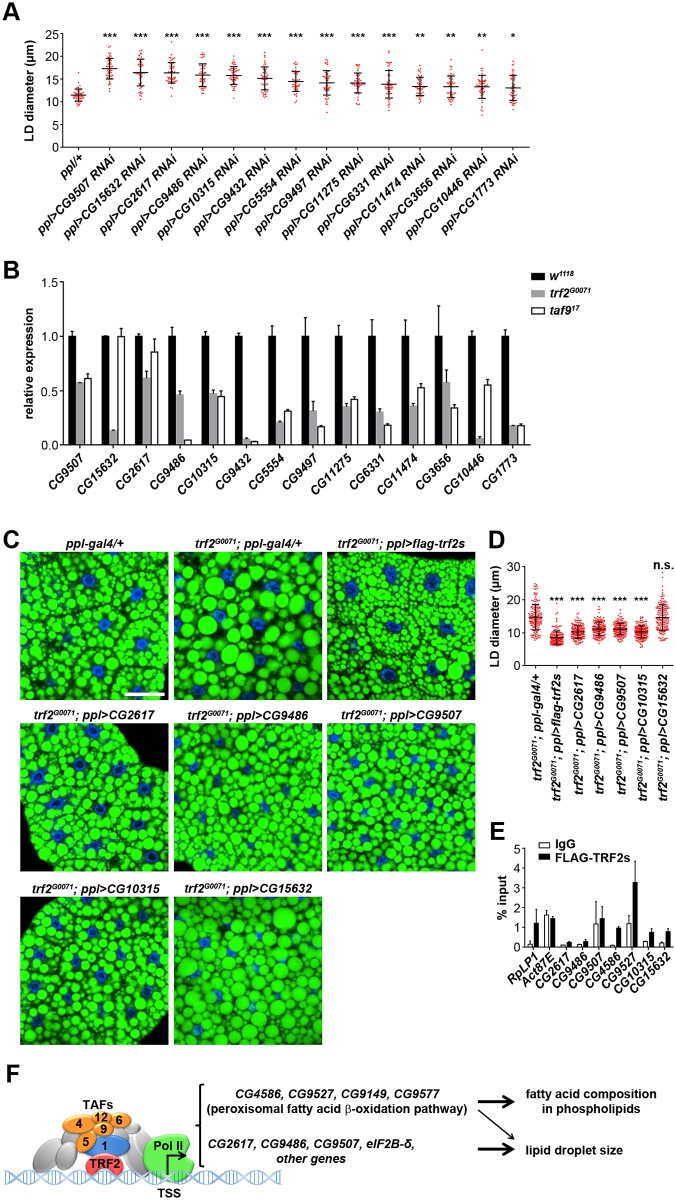
TRF2 affects LD size by directing transcription of several target genes. (A) Quantification of the diameter of the three largest LDs per cell in fat bodies of larvae with different RNAi treatments. RNAi of 14 target genes leads to enlarged LDs. Data were analyzed by one-way ANOVA with Dunnett’s multiple comparisons test. Error bars represent ±SD. ***: p < 0.001; **: p < 0.01; *: p < 0.05. (B) Relative mRNA levels of 14 target genes in fat bodies from *trf2*^*G0071*^ and *taf9*^*17*^ mutants. Error bars represent ±SD. (C) BODIPY staining of LDs in 3^rd^ instar larval fat bodies of different genetic backgrounds. Expression of *CG2617*, *CG9486*, *CG9507* and *CG10315* in the fat body partially rescues the *trf2*^*G0071*^ mutant phenotype whereas expression of *CG15632* has no rescuing effects. Scale bar represents 50 μm. (D) Quantification of LD diameter in (C). Data were analyzed by one-way ANOVA with Dunnett’s multiple comparisons test. Error bars represent ±SD. ***: p < 0.001; n.s.: nonsignificant. (E) ChIP-qPCR results showing the occupancy of FLAG-TRF2s at core-promoter regions of several target genes. *RpLP1* is a positive control while *Act87E* is a negative control. Error bars represent ±SD. (F) Schematic model depicting the regulatory mechanisms of TRF2 and TAFs on LD size and phospholipid fatty acid composition.

We next searched for other genes that may mediate the regulation of *trf2* on LD size among the 181 genes down-regulated in all three groups. We used available RNAi strains to analyze the knockdown phenotypes of the genes in larval fat body to explore their contribution to the large LD phenotype. The RNAi results showed that out of 141 genes tested, knockdown of 14 genes lead to an enlarged LD phenotype in fat bodies (Fig 7A and S3 Table). Gene expression analysis by qRT-PCR confirmed that all of these genes have decreased mRNA levels in *trf2*^*G0071*^ and *taf9*^*17*^ mutants (Fig 7B). These 14 genes have various molecular functions while some of them or their mammalian homologs have been linked to lipid metabolism based on previous studies (S4 Table). For example, *CG5554* and *CG9432* encode protein disulfide isomerases that were reported to regulate adiponectin secretion and microsomal triglyceride-transfer protein (MTP) activity [44, 45]. Moreover, these two protein disulfide isomerases were reported to associate with LDs in a previous LD proteome study [46] and knockdown of *CG9432* affects LD size and distribution in *Drosophila* S2 cells [19]. The protein Cyp4d1, encoded by *CG3656*, was also reported to associate with LDs [47]. We also found some new genes, including *CG9497*, *CG11275* and *CG11474*, which have no previous link to lipid metabolism, suggesting that they may participate in novel mechanisms in lipid storage regulation.

Among the 14 genes that caused enlarged LDs by RNAi, *CG10315*, *CG15632*, *CG2617*, *CG9486* and *CG9507* resulted in the most robust LD phenotype when knocked down (Fig 7A). To reveal the contribution of these genes to the large LD phenotype caused by *trf2* loss of function, we overexpressed them in *trf2*^*G0071*^ mutants and examined their rescuing activity. As a positive control, expression of FLAG-tagged TRF2s fully rescues the mutant LD phenotype. We found that the expression of *CG10315*, *CG2617*, *CG9486* and *CG9507* partially rescues the LD phenotype of *trf2*^*G0071*^ mutants, suggesting that these genes mediate LD size regulation by *trf2*. Expression of *CG15632* has no rescuing effects. Therefore, *CG15632* may not be a direct downstream target of *trf2* and *taf9* in LD size regulation (Fig 7C and 7D).

To investigate whether TRF2 directly regulates the expression of these genes, we performed ChIP assays on larval fat bodies expressing functional FLAG-TRF2s and examined TRF2 occupancy on the core-promoter regions of these genes. As expected, TRF2 occupies the core promoter of *RpLP1*, which is a reported TRF2 target, but not the TATA-dependent *Act87E* promoter [29]. We found that TRF2 is strongly recruited to the promoters of *CG4586*, *CG9527*, *CG10315* and *CG15632*. TRF2 is also slightly detectable on the promoters of *CG2617* and *CG9486* (Fig 7E). *CG10315* encodes the translation initiation factor eIF2B-δ. Some eIFs, such as eIF4G and eIF-4a, were reported to associate with *Drosophila* LDs [46]. Furthermore, knockdown of several eIFs, including eIF-1A, eIF-2β, eIF3ga, eIF3-S8 and eIF3-S9, causes large and condensed LDs in *Drosophila* S2 cells [19]. Taken together, these results indicate that TRF2 regulates transcription of several target genes that may play important roles in LD size regulation.

## Discussion

This study reveals a rather specific role of TRF2 and TAFs, which are general transcription factors, in regulating LD size. In addition, TRF2 and TAF9 affect phospholipid fatty acid composition, most likely through ACOX genes which mediate peroxisomal fatty acid β-oxidation (Fig 7F).

By binding to their responsive elements in target genes, specific transcription factors like SREBP, PPARs and NHR49, play important roles in lipid metabolism. It is interesting to find that the general transcription machineries, in this case TRF2 and core TAFs, also exhibit specificity in regulating lipid metabolism. In the *Drosophila* late 3^rd^ instar larval fat body, defects in *trf2* cause increased LD size, whereas mutation of the other two homologous genes, *tbp* and *trf1*, have no obvious effects on lipid storage. We also found that inactivation of *taf* genes causes a similar phenotype to *trf2* mutation, suggesting that TRF2 may associate with these TAF proteins to direct transcription of specific target genes. Moreover, we found that *trf2* mutants have large LDs at both 2^nd^ and early 3^rd^ instar larval stages, suggesting that general transcription factors are also required at early developmental stages for LD size regulation. Interestingly, *taf9* mutants have no obvious phenotype at these stages. It is possible that TAF9 may act as an accessory factor compared to promoter-binding TRF2. This is consistent with the fact that less genes are affected in *taf9* mutants than *trf2* mutants in our RNA-seq analysis. We also found that knockdown of *trf2* in larval and adult fat body leads to different LD phenotype. This may be due to different lipid storage status or different LD size regulatory mechanisms between larval and adult stages.

Our finding adds to the growing evidence supporting a specific role of general transcription factors in lipid homeostasis. For example, knockdown of RNA Pol II subunits such as RpII140 and RpII33 leads to small and dispersed LDs in *Drosophila* S2 cells [19]. Mutation in DNA polymerase delta (POLD1) leads to lipodystrophy with a progressive loss of subcutaneous fat [48]. Furthermore, TAF8 and TAF7L were reported to be involved in adipocyte differentiation [49–51]. Moreover, previous studies showed that several subunits of the Mediator complex interact with specific transcription factors and play important roles in lipid metabolism [4–10]. Added together, these lines of evidence strongly support essential and specific roles of the core/basal transcriptional machinery components in lipid metabolism.

Using RNA-seq analysis, rescue experiments and ChIP-qPCR, we identified several target genes regulated by TRF2 and TAF9. It is possible that other genes may regulate LD size but were missed in our RNA-seq analysis and RNAi screening assay because of either insufficient alterations in genes expression (lower than the twofold threshold) or low efficiency of RNAi. Among all the verified target genes of TRF2 and TAF9, *CG10315*, which strongly rescues the *trf2*^*G0071*^ mutant phenotype when overexpressed and encodes the eukaryotic translation initiation factor eIF2B-δ, may be a good candidate for further study. Although they are best known for their molecular functions in mRNA translation regulation, eIFs have been implicated in several other processes, including cancer and metabolism. For example, in yeast, eIF2B physically interacts with the VLCFA synthesis enzyme YBR159W [52]. In adipocytes, eIF2α activity is correlated with the anti-lipolytic and adipogenesis inhibitory effects of the AMPK activator AICAR [53]. In addition, given the evidence that some eIFs, such as eIF4G and eIF-4a, localize on LDs [46] and knockdown of some eIFs, including eIF-1A, eIF-2β, eIF3ga, eIF3-S8 and eIF3-S9, results in large LDs in *Drosophila* S2 cells [19], it is important to further explore the specific mechanisms of these eIFs in LD size regulation.

Although TRF2 exists widely in metazoans and shares sequence homology in its core domain with TBP, it recognizes sequence elements distinct from the TATA-box. A previous study has investigated TRF2- and TBP-bound promoters throughout the *Drosophila* genome in S2 cells and revealed that some sequence elements, such as DRE, are strongly associated with TRF2 occupancy while the TATA-box is strongly associated with TBP occupancy [28]. In our study, we also identified that DRE is significantly enriched (p-value<1e-40) in extended promoters of the 181 target genes. We further explored the distribution of TATA-boxes in the core promoters of the 181 target genes compared with all genes and found that the TATA-box is not enriched in the core promoters of TRF2 target genes. The proportion of TATA-box is 0.155 (75 of 484 isoforms) for the 181 target genes while the proportion is 0.217 (7849 of 36099 isoforms) for all genes as the background. These results suggest that TRF2 and TAF9 may regulate the expression of a subset of genes by recognizing specific sequence elements such as DRE but not the TATA-box.

We showed that expression of peroxisomal fatty acid β-oxidation pathway genes, including two acyl-CoA oxidase (ACOX) genes, *CG4586* and *CG9527*, the β-ketoacyl-CoA thiolase gene *CG9149*, and the enoyl-CoA hydratase gene *CG9577*, is regulated by TRF2 and TAF9. Lipidomic analysis indicates that in the fat body of *trf2* and *taf9* RNAi, many phospholipids, such as PA, PC, PG and PI, contain more long chain fatty acids. Furthermore, knockdown of *CG4586* and *CG9527* in the fat body also causes similar changes. These results coincide with the function of ACOX, which is implicated in the peroxisomal fatty acid β-oxidation pathway for catabolizing VLCFAs and some LCFAs. Similar to our findings, a previous study found that defective peroxisomal fatty acid β-oxidation resulted in enlarged LDs in *C*. *elegans* and blocked catabolism of LCFAs, such as vaccenic acid, which probably contributed to LD expansion in mutant worms [43]. Since overexpressing *CG4586* or *CG9527* only marginally rescues the enlarged LD phenotype of *trf2* mutants, it remains to be determined whether the increased level of long chain fatty acid-containing phospholipids contributes to LD size. Regarding the regulation of fatty acid chain length in phospholipids, a recent study reported that there was increased acyl chain length in phospholipids of lung squamous cell carcinoma accompanied by significant changes in the expression of fatty acid elongases (ELOVLs) compared to matched normal tissues. A functional screen followed by phospholipidomic analysis revealed that ELOVL6 is mainly responsible for phospholipid acyl chain elongation in cancer cells [54]. Our findings provide new clues about the regulation of fatty acid chain length in phospholipids. ELOVL and the peroxisomal fatty acid β-oxidation pathway may represent two opposing regulators in determining fatty acid chain length *in vivo*.

Previous studies have shown that TRF2 is involved in specific biological processes including embryonic development, metamorphosis, germ cell differentiation and spermiogenesis [31, 32, 55, 56]. Our results reveal a novel function of TRF2 in the regulation of specialized transcriptional programs involved in LD size control and phospholipid fatty acid composition. Since TRF2 is conserved among metazoans, its role in the regulation of lipid metabolism may be of considerable relevance to various organisms including mammals. Our findings may provide new insights into both the regulation of lipid metabolism and the physiological functions of TRF2.

## Materials and methods

### *Drosophila* stocks and husbandry

All flies were propagated on standard cornmeal food. *w*^*1118*^ was used as the wild-type control. Unless specified, *Drosophila* stocks were obtained from the Vienna *Drosophila* Resource Center, the Bloomington *Drosophila* Stock Center, the National Institute of Genetics Stock Center, the KYOTO Stock Center and the Tsinghua University RNAi Stock Center.

### Molecular biology

For gene expression constructs, the coding region of *taf9*, *trf2* and *trf2s* were inserted into the transformation vector *pUAST-attB* through the *Eco*RI and *Xho*I sites (*taf9*) or *Not*I and *Xho*I sites (*trf2l* and *trf2s*). For the FLAG-TRF2s expression construct, the *trf2s* coding region was inserted in frame into the *pUAST-attB-FLAG* vector through the *Not*I and *Xho*I sites to generate *pUAST-attB-FLAG-trf2s*. For target gene expression constructs, the coding regions of *CG9507*, *CG15632*, *CG2617*, *CG9486*, *CG10315*, *CG4586* and *CG9527* were inserted into the *pUAST-attB-FLAG* vector through the *Not*I and *Xba*I sites (*CG9507*, *CG10315*, *CG4586* and *CG9527*) or *Not*I and *Bgl*II sites (*CG15632*, *CG2617* and *CG9486*). The coding region of all genes was amplified from *w*^*1118*^-derived cDNA.

### Quantitative RT-PCR

Total RNA was isolated from wandering 3^rd^ instar larval fat body using Trizol reagent (Invitrogen) and cDNA was generated using a Superscript II reverse transcriptase kit (Invitrogen). qRT-PCR experiments were performed with a Stratagene Mx3000P system (Agilent) using Transstart Green qPCR superMix (Transgen). Relative levels of expression were normalized to *rp49* in the same sample.

### Generation of *taf9* mutant alleles

We used imprecise P-element excision to generate *taf9* mutant alleles. The starting P-element *P{GT1}e(y)1*^*BG00948*^ harbors the *white* (*w*^+^) marker gene. The *TM3Δ2–3* males which provide the transposase were mated to *e(y)1*^*BG00948*^ virgin females. The F1 males (*e(y)1*^*BG00948*^; *TM3Δ2–3*) were then crossed with *FM7i* virgin females. The F2 progeny females were screened for site-directed P-element excision by the loss of the eye color marker *w*^+^ and individually balanced to establish stocks. Two *taf9* deletion mutants were identified by PCR from 350 balanced single-cross stocks.

### Staining and microscopy

For LD staining, wandering 3^rd^ instar larvae were dissected in PBS and the fat body was fixed in 4% paraformaldehyde for 30 min at room temperature. Tissues were then rinsed twice with PBS, incubated for 30 min in either a 1:500 dilution with PBS of 1 mg/ml BODIPY 493/503 (Invitrogen) or a 1:100 dilution with PBS of LipidTOX Deep Red (Invitrogen) and then rinsed twice with PBS. 2 ng/μl DAPI was used to stain nuclei. Stained samples were mounted in 75% glycerol for microscopy analysis. All images were taken using a confocal microscope. To quantify LD size, the diameters of 160 LDs (larger than 5 μm that can be accurately measured) from 20 fat cells, or the three largest LDs in each of 16 fat cells, were measured by NIS-Elements BR 3.0 software.

### RNA-seq analysis

Total RNA was isolated from wandering 3^rd^ instar larval fat body using an RNeasy lipid tissue kit (Qiagen) following the manufacturer’s instruction and 10 μg of total RNA was used to prepare Poly-A RNA-seq libraries. Samples were sequenced using an Illumina HiSeq 2000 sequencer at BGI TechSolutions Co., Ltd. (BGI-Tech). Sequenced reads were aligned to reference sequences using SOAPaligner/SOAP2 [57]. Genes expressed with >twofold difference and FDR (False Discovery Rate) ≤0.001 were considered as differentially expressed for scatter plot representations and for GO term analysis using GOseq [58]. The raw sequencing data have been submitted to the Genome Sequence Archive (GSA) database with the accession number PRJCA000264.

### ChIP assay and quantitative PCR

Chromatin preparation and immunoprecipitation was performed as previously described with some modifications [59]. Wandering 3^rd^ instar larvae were dissected and fat bodies expressing FLAG-TRF2s were collected and treated with 1% formaldehyde in PBS for 15 min at room temperature for crosslinking. The reaction was then quenched with 125 mM glycine for 5 min and the treated samples were washed twice with PBS containing 1 mM PMSF and Protease Inhibitor Cocktail (Roche). The samples were homogenized with a cordless motor in FA buffer and the chromatin was sheared to a size range of 100–1000 bp by sonication. Cellular debris and floating lipids were removed by several centrifugation steps at 13,000 rpm for 15 min at 4°C and the supernatant was used for chromatin immunoprecipitation (ChIP). Anti-FLAG antibody (Sigma) and protein A- and protein G-agarose beads (Millipore) were used in the immunoprecipitation experiments. Anti-mouse IgG antibody (Promega) was used as the negative control. Eluted DNA was purified using a PCR purification kit (Qiagen) and then quantified by quantitative PCR. Sequences of primers used for qPCR can be found in S5 Table.

### Lipidomic analysis

Lipids were extracted from wandering 3^rd^ instar larval fat body as previously described [60]. The lipidomic analyses were carried out on an analytical system comprising an Agilent HPLC 1260 coupled with a SCIEX 5500 QTRAP. Separation of individual classes of polar lipids by normal phase HPLC was carried out using a Phenomenex Luna 3u silica column (i.d. 150x2.0 mm). Multiple reaction monitoring (MRM) transitions were set up for quantitative analysis of various polar lipids. Individual lipid species were quantified by referencing to spiked internal standards. PC-14:0/14:0, LPC-C20, PE-14:0/14:0, PS-14:0/14:0, PA-17:0/17:0, PG-14:0/14:0 were obtained from Avanti Polar Lipids and dioctanoyl phosphatidylinositol (PI, 16:0-PI) was obtained from Echelon Biosciences, Inc. Separation of glycerol lipids (DAG and TAG) by reverse phase HPLC/ESI/MS/MS was carried out on a Phenomenex Kinetex 2.6μ-C18 column (i.d. 4.6x100mm). Using neutral loss-based MS/MS techniques, the levels of TAG were calculated as relative contents to the spiked d5-TAG 48:0 internal standard (CDN Isotopes), while DAG species were quantified using 4ME 16:0 Diether DG as an internal standard (Avanti Polar Lipids). Free cholesterols and ergosterols were analyzed using HPLC/APCI/MS/MS with the corresponding d6-Cho (CDN Isotopes) as the internal standard.

### Motif analysis

Promoters of target genes were analyzed using the motif discovery software HOMER [61]. We defined two types of promoter in the analysis. The core promoter is the DNA sequence from -100 to +50 (relative to +1 of the TSS) while the extended promoter is from -500 to +50. When using HOMER, we set the corresponding regions of all genes in the *Drosophila* genome as the background sequences. The significantly enriched motifs were selected by the criterion of q-value <0.05 and fold change >1.5. The q-value is provided by the HOMER output and the fold change is defined as the ratio between the number of target sequences that contain the motif and the number of background sequences that contain the motif.

## Supporting information

S1 Table RNAi phenotype of *Drosophila taf* genes. A summary of the RNAi phenotype and fly strains of *taf* genes is shown.
(DOCX)

S2 Table RNAi strains of *CG4586* and *CG9527* used in the study. (DOCX)

S3 Table 141 genes tested in the RNAi screen of *trf2* and *taf9* target genes. (DOCX)

S4 Table *trf2* and *taf9* target genes with LD phenotype. The molecular functions of *trf2* and *taf9* target genes with LD phenotype are listed in the table.
(DOCX)

S5 Table Primers for target gene core promoters used in ChIP-qPCR assays. (DOCX)

S1 Fig The LD phenotype of *trf2* and *taf9* mutants at 2^nd^ and early 3^rd^ instar larval stages. BODIPY staining of LDs in the fat body of 2^nd^ instar (A) and early 3^rd^ instar (B) larvae from different backgrounds. There is no obvious difference between *taf9*^*17*^ mutants and *w*^*1118*^ control, while *trf2* mutants have large LDs. Scale bar represents 20 μm (A) and 50 μm (B), respectively.
(TIF)

S2 Fig TRF2 regulates LD size in adult fat body. BODIPY staining of LDs in the fat body of 7-days old female adults. Knockdown of *trf2* leads to more small LD. Scale bar represents 20 μm.
(TIF)
