# Supplementary material for: Drosophila TRF2 and TAF9 regulate lipid droplet size and phospholipid fatty acid composition
Source: PLoS Genet. 2017 Mar 8;13(3):e1006664. doi: 10.1371/journal.pgen.1006664 (PMC5362240; doi:10.1371/journal.pgen.1006664)
Supplement: S1 Table — A summary of the RNAi phenotype and fly strains of taf genes is shown. (DOCX) [file pgen.1006664.s001.docx]

S1 Table. RNAi phenotype of *Drosophila taf* genes.

|  | CG No. | Symbol | Phenotype | RNAi strain |
| --- | --- | --- | --- | --- |
| *taf* genes with enlarged LD phenotype | *CG17603*  *CG5444*  *CG7704*  *CG32211*  *CG6474*  *CG17358* | *taf1*  *taf4*  *taf5*  *taf6*  *e(y)1*/*taf9*  *taf12* | +  ++  +  +  +  + | KK100418, GD4594  KK101958  GD4096  KK101106, GD10817  HMS00336  GD4149 |
| *taf* genes with no obvious LD phenotype | *CG6711*  *CG2009*  *CG6577*  *CG10390*  *CG2670*  *CG7128*  *CG2859*  *CG3069*  *CG4079*  *CG10756* | *taf2*  *bip2*/*taf3*  *can*/*taf5L*  *mia*/*taf6L*  *taf7*  *taf8*  *taf10*  *taf10b*  *taf11*  *taf13* | no  no  no  no  no  no  no  no  no  no | GD4084  KK102931  GD4085  KK107112  GD4592  KK102373  KK110439, GD4727  KK102240, GD4055  KK107818, GD4059  KK112084 |

+: average size of LDs is between 9μm (for *ppl-Gal4* control) to 12μm.

++: average size of LDs is over 12μm.
