# Supplementary material for: Drosophila TRF2 and TAF9 regulate lipid droplet size and phospholipid fatty acid composition
Source: PLoS Genet. 2017 Mar 8;13(3):e1006664. doi: 10.1371/journal.pgen.1006664 (PMC5362240; doi:10.1371/journal.pgen.1006664)
Supplement: S2 Table — (DOCX) [file pgen.1006664.s002.docx]

S2 Table. RNAi strains of *CG4586* and *CG9527* used in the study.

| CG No. | RNAi strain |
| --- | --- |
| *CG4586* | 4586R-2  4586R-3  HMJ21973  GD11363 |
| *CG9527* | HMJ21884  KK105295  GD14239 |
