# Supplementary material for: Drosophila TRF2 and TAF9 regulate lipid droplet size and phospholipid fatty acid composition
Source: PLoS Genet. 2017 Mar 8;13(3):e1006664. doi: 10.1371/journal.pgen.1006664 (PMC5362240; doi:10.1371/journal.pgen.1006664)
Supplement: S3 Table — (DOCX) [file pgen.1006664.s003.docx]

S3 Table. 141 genes tested in the RNAi screen of *trf2* and *taf9* target genes.

| CG No. | Symbol | RNAi strain |
| --- | --- | --- |
| *CG1236* |  | TH02389.N |
| *CG1391* | *sol* | JF03400 |
| *CG1401* | *Cul5* | TH01798.N |
| *CG1732* | *Gat* | JF03358 |
| *CG1773* |  | 1773R-1 |
| *CG2045* | *Ser7* | HMS02485 |
| *CG2201* |  | GL00323 |
| *CG2617* |  | 2617R-1 |
| *CG2679* | *gol* | JF03213 |
| *CG2816* |  | HMJ21938, GL01212 |
| *CG3006* | *Fmo-1* | 3006R-1 |
| *CG3327* | *E23* | JF02151 |
| *CG3376* |  | HMS03021 |
| *CG3394* |  | 3394R-2 |
| *CG3421* | *RhoGAP93B* | HMS01440, JF01660 |
| *CG3571* | *KLHL18* | 3571R-1 |
| *CG3589* |  | 3589R-2 |
| *CG3656* | *Cyp4d1* | 3656R-1 |
| *CG3700* |  | 3700R-2 |
| *CG3714* |  | 3714R-2 |
| *CG3879* | *Mdr49* | HMS00400 |
| *CG4073* |  | 4073R-3 |
| *CG4080* |  | 4080R-2 |
| *CG4630* |  | HMJ23028 |
| *CG4900* | *Irp-1A* | HMJ22054 |
| *CG4927* |  | HMJ23184 |
| *CG5151* |  | HMJ21912 |
| *CG5270* |  | 5270R-2 |
| *CG5489* | *Atg7* | HMS01358, JF02787 |
| *CG5554* |  | 5554R-1 |
| *CG5618* |  | JF02627 |
| *CG5958* |  | 5958R-1 |
| *CG5989* |  | HMJ21976 |
| *CG6012* |  | 6012R-1 |
| *CG6163* |  | 6163R-1 |
| *CG6201* |  | TH02025.N |
| *CG6225* |  | TH01907.N |
| *CG6331* | *Orct* | 6331R-2 |
| *CG6741* | *a* | TH02162.N |
| *CG6762* |  | TH05251.N |
| *CG6928* |  | 6928R-1 |
| CG No. | Symbol | RNAi strain |
| *CG7227* |  | TH01702.N |
| *CG7242* | *Spc25* | 7242R-2 |
| *CG7408* |  | 7408R-2 |
| *CG7437* | *mub* | HMS00189 |
| *CG7446* | *Grd* | HMS01853 |
| *CG7837* |  | TH03536.N |
| *CG8024* | *Rab32* | JF02836, HMS01870 |
| *CG8112* |  | 8112R-2 |
| *CG8213* |  | HMJ22360 |
| *CG8475* |  | HMS01249 |
| *CG8643* | *rgr* | TH01999.N, JF02216 |
| *CG8727* | *cyc* | JF02185 |
| *CG8902* | *Nuf2* | HMS01616 |
| *CG9200* | *Atac1* | TH03845.N |
| *CG9220* |  | HMC03343 |
| *CG9270* |  | HMS00999 |
| *CG9432* | *l(2)01289* | 30444R-1 |
| *CG9444* |  | 9444R-2 |
| *CG9456* | *Spn42Dd* | HMJ21872 |
| *CG9485* |  | HMS01321 |
| *CG9486* | *AANATL2* | 9486R-1 |
| *CG9492* |  | TH02057.N |
| *CG9497* |  | 9497R-2 |
| *CG9507* |  | 9507R-1 |
| *CG9520* | *C1GalTA* | 9520R-2 |
| *CG9610* | *Poxm* | JF02321 |
| *CG9897* |  | HMC04107 |
| *CG9927* | *Art6* | HMJ22306 |
| *CG10033* | *for* | JF01449 |
| *CG10108* | *phyl* | JF03369 |
| *CG10157* | *GILT2* | HMJ21033 |
| *CG10186* | *Hasp* | 10186R-1 |
| *CG10315* | *eIF2Bdelta* | 10315R-1 |
| *CG10424* |  | 10424R-1 |
| *CG10446* | *Sidpn* | 10446R-1 |
| *CG10483* |  | HMS01023 |
| *CG10521* | *NetB* | HMS01177 |
| *CG10755* | *Cyp4ae1* | TH04967.N |
| *CG10946* | *dpr14* | JF03305 |
| *CG11129* | *Yp3* | TH03152.N |
| *CG11156* | *mus101* | 11156R-1 |
| *CG11236* |  | TH04297.N |
| *CG11275* |  | 11275R-1 |
| CG No. | Symbol | RNAi strain |
| *CG11474* |  | 11474R-1 |
| *CG11491* | *br* | HMS00042, JF02585 |
| *CG12143* | *Tsp42Ej* | JF03325 |
| *CG12272* | *Strumpellin* | TH02375.N |
| *CG12789* | *santa-maria* | HMS01405, JF03227 |
| *CG12926* |  | 12926R-1 |
| *CG13229* |  | JF03355 |
| *CG13334* |  | 13334R-1 |
| *CG13388* | *Akap200* | HMJ02109 |
| *CG13434* | *Nnf1a* | 13434R-1 |
| *CG13575* |  | JF01845 |
| *CG13594* |  | HMS02139 |
| *CG13675* |  | HMJ21266 |
| *CG13796* |  | HMJ22974 |
| *CG13897* | *hng3* | 13897R-1 |
| *CG14106* |  | HMJ30126 |
| *CG14629* |  | 14629R-1 |
| *CG14902* | *Decay* | TH02088.N |
| *CG14974* |  | 14974R-1 |
| *CG15632* | *Taf12L* | 15632R-1 |
| *CG15822* |  | 15822R-1 |
| *CG16952* |  | HMJ21605 |
| *CG17058* | *Peritrophin-A* | HMJ22580 |
| *CG17104* |  | HMS02618 |
| *CG17224* |  | 17224R-3 |
| *CG17577* | *Cyp9h1* | HMJ23527 |
| *CG17739* |  | JF03198 |
| *CG18023* | *Eip78C* | JF02258 |
| *CG18146* | *NimC2* | JF01980 |
| *CG18287* | *ppk19* | JF01928 |
| *CG30059* |  | 30059R-1 |
| *CG30152* | *galla-1* | 30152R-1 |
| *CG30359* | *Mal-A5* | HMJ21242 |
| *CG30456* |  | JF01726 |
| *CG31100* |  | 11976R-1 |
| *CG31102* |  | 31102R-1 |
| *CG31344* |  | 6572R-1 |
| *CG31549* |  | HMJ23049 |
| *CG31760* |  | TH02067.N |
| *CG31904* |  | 31904R-2 |
| *CG31958* |  | JF01142 |
| *CG32082* | *IRSp53* | TH01830.N |
| *CG32180* | *Eip74EF* | JF02515 |
| CG No. | Symbol | RNAi strain |
| *CG32232* |  | TH05072.N |
| *CG32251* | *Claspin* | HMS00772 |
| *CG32695* |  | HMJ21616 |
| *CG32774* | *Muc4B* | HMJ23062 |
| *CG32786* |  | HMJ23988 |
| *CG33233* |  | 12036R-1 |
| *CG33547* | *Rim* | TH01596.N, JF02610 |
| *CG34274* |  | HMJ23133 |
| *CG34340* | *Drgx* | HMJ22871 |
| *CG42330* | *Dscam4* | TH02072.N |
| *CG42748* |  | HMJ22972 |
| *CG43173* |  | HMS02369 |
| *CG43368* | *cac* | JF02572 |
| *CG43934* | *Hr4* | HMJ21497 |
