# Supplementary material for: Drosophila TRF2 and TAF9 regulate lipid droplet size and phospholipid fatty acid composition
Source: PLoS Genet. 2017 Mar 8;13(3):e1006664. doi: 10.1371/journal.pgen.1006664 (PMC5362240; doi:10.1371/journal.pgen.1006664)
Supplement: S4 Table — The molecular functions of trf2 and taf9 target genes with LD phenotype are listed in the table. (DOCX) [file pgen.1006664.s004.docx]

S4 Table. *trf2* and *taf9* target genes with LD phenotype.

| CG No. | Symbol | Function |
| --- | --- | --- |
| *CG1773* |  | serine-type endopeptidase activity |
| *CG2617* |  | RING finger domain protein, ortholog of human RNF26 |
| *CG3656* | *Cyp4d1* | cytochrome P450 family member |
| *CG4586* |  | acyl-CoA oxidase activity, fatty acid β-oxidation in peroxisome |
| *CG5554* |  | protein disulfide isomerase activity |
| *CG6331* | *Orct* | organic cation transmembrane transporter activity |
| *CG9432* | *l(2)01289* | protein disulfide isomerase activity |
| *CG9486* | *AANATL2* | aralkylamine N-acetyltransferase activity |
| *CG9497* |  | unknown |
| *CG9507* |  | metalloendopeptidase activity |
| *CG9527* |  | acyl-CoA oxidase activity, fatty acid β-oxidation in peroxisome |
| *CG10315* | *eIF2B-δ* | translation initiation factor activity |
| *CG10446* | *Sidpn* | ortholog of mouse HES1 |
| *CG11275* |  | unknown |
| *CG11474* |  | unknown |
| *CG15632* | *Taf12L* | testis-specific TBP-associated factor |
