# Supplementary material for: Drosophila TRF2 and TAF9 regulate lipid droplet size and phospholipid fatty acid composition
Source: PLoS Genet. 2017 Mar 8;13(3):e1006664. doi: 10.1371/journal.pgen.1006664 (PMC5362240; doi:10.1371/journal.pgen.1006664)
Supplement: S5 Table — (DOCX) [file pgen.1006664.s005.docx]

S5 Table. Primers for target gene core promoters used in ChIP-qPCR assays.

| Gene | primer site * | Sequence (from 5’ to 3’) |
| --- | --- | --- |
| *RpLP1* forward | -117 ~ -97bp | TAATCTCGGCAGTTTGAACG |
| *RpLP1* reverse | +14 ~ +34bp | CACGCTGGTCAAAATCCTAA |
| *Act87E* forward | -74 ~ -54bp | GTGCGTGCGGAAAATATCTA |
| *Act87E* reverse | +68 ~ +88bp | GGAATTGGTGGATGTGGAGT |
| *CG2617* forward | -53 ~ -33bp | TGACGCTACCTTATCGCAAC |
| *CG2617* reverse | +52 ~ +74bp | GGCTCATACTGTCCTCGAATTA |
| *CG4586* forward | -100 ~ -79bp | TCGGATCACTGATAACCAACC |
| *CG4586* reverse | +28 ~ +49bp | CTACGGGATTTCCCAACTTCT |
| *CG9486* forward | -103 ~ -83bp | AAGCTCAGAACGGAGACGAT |
| *CG9486* reverse | +74 ~ +94bp | AAGAAGTGCACTGCGAGAAA |
| *CG9507* forward | -123 ~ -101bp | CAAGTGGTCAAGTACTGGGTGT |
| *CG9507* reverse | +46 ~ +66bp | ATATGAGCCAGGCCACTAGG |
| *CG9527* forward | -176 ~ -156bp | AAATTACGCGCCAGACCTAC |
| *CG9527* reverse | +38 ~ +60bp | CGTGACTTAAGCCATGAGAAAG |
| *CG10315* forward | -107 ~ -87bp | AGCTATGGGAATGCCAACTC |
| *CG10315* reverse | +48 ~ +68bp | CTCGAAAGTTTTGCTCACGA |
| *CG15632* forward | -110 ~ -90bp | AAGACACAGGACCCGAAAAG |
| *CG15632* reverse | +60 ~ +80bp | CGACGATTTATCGCTGTCAT |

*: The primer site is relative to the transcription start site (TSS) at +1.
