# Supplementary figures and images for: Drosophila TRF2 and TAF9 regulate lipid droplet size and phospholipid fatty acid composition
Source: PLoS Genet. 2017 Mar 8;13(3):e1006664. doi: 10.1371/journal.pgen.1006664 (PMC5362240; doi:10.1371/journal.pgen.1006664)

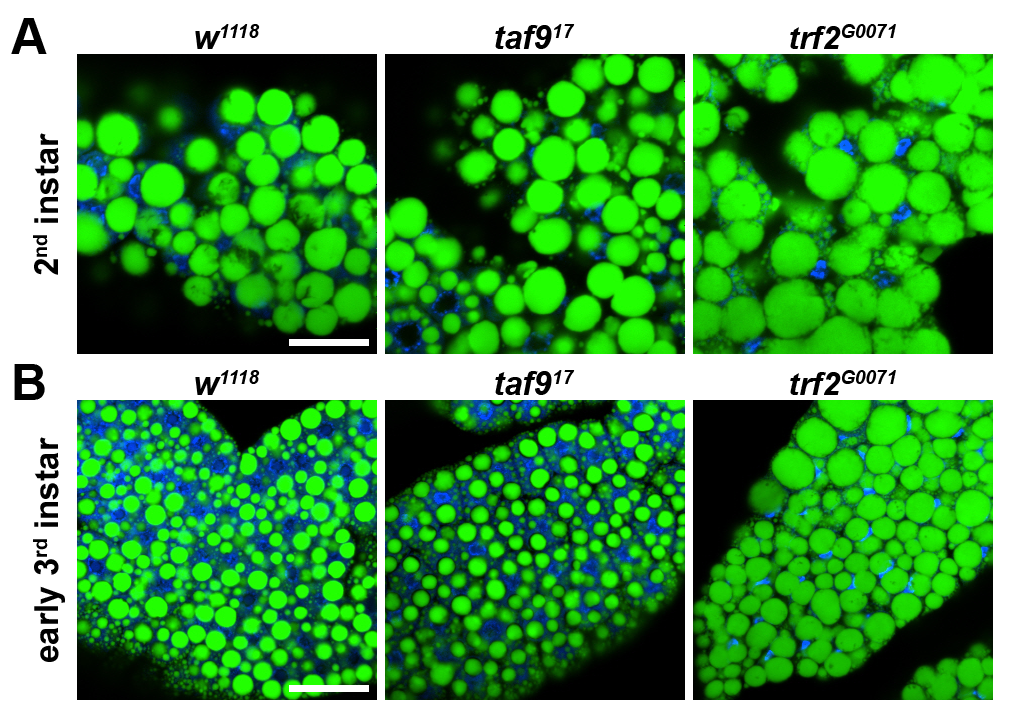

Supplement: S1 Fig — BODIPY staining of LDs in the fat body of 2nd instar (A) and early 3rd instar (B) larvae from different backgrounds. There is no obvious difference between taf917 mutants and w1118 control, while trf2 mutants have large LDs. Scale bar represents 20 μm (A) and 50 μm (B), respectively. (TIF) [file pgen.1006664.s006.tif]

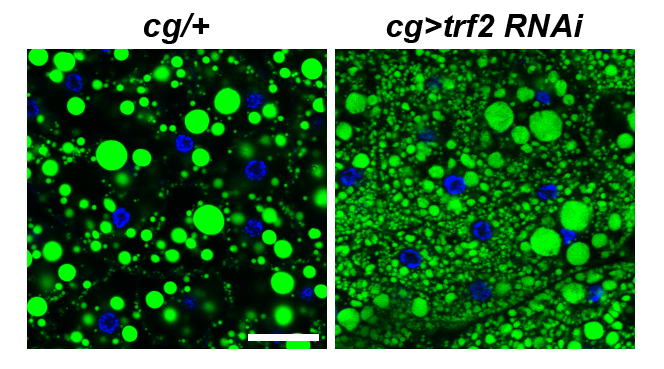

Supplement: S2 Fig — BODIPY staining of LDs in the fat body of 7-days old female adults. Knockdown of trf2 leads to more small LD. Scale bar represents 20 μm. (TIF) [file pgen.1006664.s007.tif]
